# Web‐Based Sustainable Detection and Treatment Recommendation System for Wheat Plant Diseases Using Convolutional Neural Networks

**DOI:** 10.1002/fsn3.71445

**Published:** 2026-01-14

**Authors:** Nergis Gulzar Abbasi, Aiman Khan Nazir, Sadia Ali, Hamza Iqbal, Atif Ali, Asghar Ali Shah, Sagheer Abbas, Muhammad Adnan Khan

**Affiliations:** ^1^ University Institute of Information Technology Pir Mehr Ali Shah Arid Agriculture University Rawalpindi Pakistan; ^2^ RMC Multimedia University Cyberjaya Malaysia; ^3^ Department of Computer Science Kateb University Kabul Afghanistan; ^4^ Department of Computer Science Prince Mohammad Bin Fahd University Alkhobar Saudi Arabia; ^5^ Department of Software, Faculty of AI and Software Gachon University Seongnam‐si South Korea

**Keywords:** CNN, convolutional neural network, rust diseases, wheat disease detection, wheat disease treatment

## Abstract

Wheat, being a major staple crop worldwide, is often attacked by rust diseases, which cause severe yield losses. The early detection and diagnosis of fungal infections, yellow rust, and brown rust are critical in minimizing their consequences. A web‐based system based on a Convolutional Neural Network (CNN) was developed for the quick identification and classification of wheat plant diseases. The diseases that we examine in wheat plants are brown rust (BR) and yellow rust (YR), and healthy plants are classified in the third category. A dataset of labeled images of YR, BR, and healthy wheat plants was used to train the CNN. The model achieved a remarkable 96% classification accuracy. In addition to disease diagnosis, a recommendation module that gives advice on proper treatment based on disease names or symptoms is also provided. This twofold functionality allows for timely disease management and identification and facilitates the treatment of other wheat diseases besides rust diseases. Integrating the trained CNN model into an intuitive web application makes it user‐friendly for end users, notably farmers, to have a practical tool in protecting wheat crops.

## Graphical abstract

A comprehensive dataset of wheat crop images, including healthy plants and samples affected by YR and BR diseases, was developed from public sources. A Convolutional Neural Network (CNN) model was trained and optimized for accurate detection and classification of wheat diseases. The trained model was integrated into a user‐friendly web application, enabling farmers and agricultural stakeholders to quickly identify wheat diseases and receive effective treatment recommendations for crop protection and management.

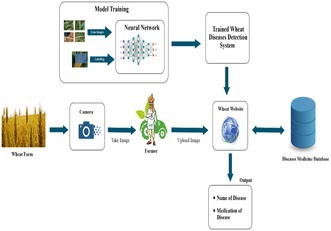

## 1 Introduction

Agriculture is important to economies of countries like Pakistan that aren't as well off as others. Agriculture is a main part of Pakistan's income. The agriculture industry makes up 21% of Pakistan's GDP and grows by 2.7% each year (Abade et al. 2021; Azam and Shafique 2017; Bose and Kiran 2021). 44% of the labor force finds work in the agricultural sector, while 62% of rural residents depend on it for their primary source of income. A distinct area of the overlap of our life ways and economic development is in the field of agriculture. The agricultural sector takes some roles in the economic systems of all countries including the provision of food security, curbing poverty, industrialization, and economic growth, especially in the developing world (Abade et al. 2021; Abdul‐Mumin 2022; Awan et al. 2021). In Pakistan, the agricultural sector depends on the agricultural crops like sugarcane, rice, maize, wheat, and cotton. The crops are important in value added in agricultural activities and they account for about 24% of the overall value added. Moreover, they also have a contribution towards the Gross Domestic Product (GDP) in the country of 4.67%. Therefore, the crops are major pillars of the Pakistani agricultural sector. There are minor crops such as Bajra, Jowar, mash, and gram which contribute about 2.25% to GDP and about 11.36% value added to the total agricultural industry (Abade et al. 2021; Azam and Shafique 2017; Bose and Kiran 2021).

One of the major grain crops that are widely grown and are the leading staple foods within the country is wheat. The growth and affluence of wheat have a significant impact on the health of people. Wheat is particularly important in world food security, with most individuals of the world consuming it, over 50% (Cao et al. 2019). The contribution of wheat to GDP is relatively high and amounts to 3.1%, with 14.4% value added in the agricultural sector. The expected rate of growth of a wheat crop is 11.7% (Azam and Shafique 2017; Figueroa et al. 2018; Jahan et al. 2020). Nevertheless, wheat diseases have a serious threat to the safety of wheat production (Ma et al. 2019). Three most observable wheat diseases are rusts, blotches, and head blight/scabs (Deng et al. 2022; Moshou et al. 2004; Zheng et al. 2018). Other diseases which usually go undetected but pose a major threat to the production of grain include wheat blast (WB) and spot blotch (SB) (Figueroa et al. 2018).

Rust infections have consistently hampered worldwide wheat production since wheat was domesticated and continue to be a threat to the world's wheat supply (Roelfs et al. 1992). The rusts are the most destructive. Rust diseases, specifically YR and BR, are fungal infections that can have a significant negative influence on wheat yield. They result in rust‐like lesions on wheat plants' leaves, stems, and grains, which lowers yield and quality (Sood and Singh 2020). Puccinia striiformis f. sp. tritici (Pst) causes YR, which results in explosive disease outbreaks and local epidemics (Moshou et al. 2004; Zhang et al. 2019; Zheng et al. 2018). YR can cause a harvest loss or a more than 40% decrease in wheat yield during epidemic years (Chen 2005; Chen et al. 2022). A fungus called Puccinia triticina is the cause of BR. The leaves are affected by BR (Deng et al. 2022; Sood and Singh 2020). The rust infections, to put it simply, fall under the category of wheat fungal disease. As a result of the widespread use of pesticides, it is believed that fungal diseases are spreading in wheat crops (Haider et al. 2021). In other words, during an infected season, these pesticides do not disrupt wheat. A difficulty encountered was the limited availability of fungicides and protective gear in certain regions of the country. This lack of supply did not only impede pest management in good time but also resulted in serious health and safety concerns to farmers (Mi et al. 2020). Moreover, many farms need to be sprayed more often, and detecting infectious diseases in a timely manner will allow reducing the decline in harvest. This brings in the significance of the identification of wheat diseases to maximize the yield and quality of wheat and assist farmers in locating and obtaining rust‐covered leaves. Hence, prevention and control of wheat diseases and minimization of losses can only be effectively achieved through closely observing their occurrence.

Identification of wheat diseases is done either by trained evaluators or by computer‐aided technology. Wheat fungal disease detection under the observation of an expert evaluator requires a lot of time and money (Picon et al. 2019). Further, it has major disadvantages, which include low coverage (Zheng et al. 2018). Through the aspects of computer vision, it becomes easy to identify the presence of fungal diseases in wheat. Utilizing these techniques simplifies wheat disease identification, resulting in reduced costs and time compared to relying on experienced evaluators. With the rapid advancement of deep learning technology in recent years, individuals have started experimenting with a variety of machine vision applications for the detection of insect pests and agricultural diseases. The task and prior knowledge must be taken into account while choosing the right features in traditional machine vision algorithms. These characteristics often include an image's color, form, and texture. The manual design is the foundation for feature extractors. The procedure is laborious and challenging. Additionally, feature extractors are incapable of generalization. On the other hand, Deep Learning Models (DLM) can modify weight parameters and create an appropriate feature extractor. The procedure is quite convenient and effective. Additionally, feature extractors have greater generalization capabilities, which can successfully address the drawbacks of traditional machine vision techniques.

Artificial intelligence and DLM can automatically learn deep features from an image dataset, due to which they are increasingly being used in agricultural research; also, their levels of precision and speed surpass those of conventional algorithms (Z. Chen et al. 2022). CNN is a widely used DLM architecture. It was designed specifically to operate with image and video recognition, etc. It is used in various areas such as identifying plant diseases (Astani et al. 2022; Garg and Datta 2013; Tugrul et al. 2022; Upadhye et al. 2022), health care recommendation systems (Shaikh et al. 2022), detection of weeds (Khan et al. 2022), and identification of pests (Cheng et al. 2021), etc.

Lo et al. emphasize the importance of logical structure, clarity, and coherence in scholarly communication to enhance manuscript quality and publication success. The authors highlight that well‐organized presentation, transparent reporting, and adherence to journal guidelines are critical skills for effective academic writing. Their findings underscore that developing these competencies improves the readability, credibility, and overall impact of scientific publications (Lo 2022).

In our research paper, we utilized a CNN to detect Wheat Plant Diseases (WPD). The model is capable of classifying YR, BR, and healthy plants. Additionally, we developed a web‐based application that suggests treatments for diseases and provides information on wheat diseases based on their symptoms. The application can also identify corresponding diseases and their remedies if a user inputs symptoms. The web‐based application not only offers symptoms and treatments for YR and BR but also for various other diseases, including Powdery Mildew (PM), Septoria Leaf Blotch (SLB), Tan Spot (TS), Head Blight (HB) (Scab), Loose Smut (LS), Karnal Bunt (KB), Root Rot (RR), Leaf Blight (LB), Barley Yellow Dwarf Virus (BYDV), Wheat Soilborne Mosaic Virus (WSBMV), and Wheat Streak Mosaic Virus (WSMV).

The main contributions of research are as follows:

- The construction of a comprehensive data repository was undertaken with great care, wherein a collection of photographs of wheat harvests was methodically compiled. The photos were carefully selected from publicly accessible sources, which include examples of YR and BR diseases and healthy plants.
- CNN, which is a sophisticated approach to deep learning, was used in the case of wheat disease detection. Our CNN model was highly effective and trained and optimized to identify and classify various forms of wheat diseases represented in plant images with high accuracy.
- Implementation of the model in a web‐based application whose interface is user‐friendly. To make it easier to use and more accessible to the farmers and other players in the agricultural industry, we were able to place a trained CNN model within a web‐based application, which is easy to use and navigate. This program is a highly efficient tool for early detection of wheat diseases and gives useful treatment recommendations to help in the control and protection of crops.

Overall, our research is a worthy contribution to the development of a well‐developed online tool that will be particularly useful in terms of disclosing WPD and giving pharmacological prescriptions. The holistic approach combines information curation, modern deep learning techniques, and user‐friendly design to become a valuable resource to the agricultural community.

The trained CNN model is easy to use because it can be integrated into an intuitive web application, which is accessible to end users, especially farmers, to have a useful tool in safeguarding wheat crops. The problem of fluctuating natural light, camera position, and image quality in the real‐world field applications is, however, recognized to be a limitation on system robustness and needs to be constantly improved to achieve optimum effectiveness.

The rest of the paper sections will be organized in the following way: Literature on wheat disease detection is analyzed in Section 2. Section 3 discusses the suggested approach. Section 4 talks about the results and justification of the methodology suggested. Section 5 gives a conclusion and suggestions to pursue research.

## 2 Related Work

When performed by humans, identification of wheat diseases is prone to error. In recent years, scientists have classified wheat diseases using DLM. However, each researcher has employed distinct image datasets and disease detection (DD) methodologies. There are two types of wheat disease classification research: the first type entails manual feature extraction and classification using machine learning algorithms, whereas the second type focuses on automatic classification using DLM. This section provides an in‐depth analysis of both categories and summarizes them in Table 1.

**TABLE 1:** Outcome of literature review.

Studies	Feature extraction technique	Classification technique	Classification labels	Accuracy	Treatment recommendation
Xie et al. (2016)	Gray level co‐occurrence matrix (GLCM)	SVM RVM	YR and PM	77.78% 88.89%	No
Römer et al. (2011)	Principal Component Analysis (PCA)	SVM ANN Decision trees	BR and health plant	93.05%	No
Su et al. (2018)	Feature Scoring Algorithms (Mutual Information)	Random forest	YR and healthy plant	89.4%	No
Wang et al. (2015)	Wavelet packet analysis	k‐nearest neighbors (KNN) SVM	YR and BR	92% 68.67%	No
Majumdar et al. (2015)	C‐means Clustering	ANN	Snow molds (SM), YR, SLB, and PM.	84.8%	No
Mi et al. (2020)	Automatic Extraction	CNN Resnet‐50 Alexnet VGG‐16	SB, YR, black rust and LS	97.20% 97.12% 95.10% 96.72%	No
Pryzant et al. (2017)	Automatic Extraction	RNN DNN	SB YR, black rust, and LS	—	No
Zhang et al. (2019)	Automatic Extraction	DCNN	YR	85%	No

### 2.1 Machine Learning Methods

Several researchers use various machine learning techniques to classify wheat illnesses in a variety of applications (Gupta et al. 2019). The process of extracting features from photographs is performed manually by application of feature extraction techniques (Jahan et al. 2020). The practice of manually extracting features presents a substantial obstacle within the domain of machine learning, thereby exerting a notable influence on the classification procedure. The utilization of feature extraction techniques is of utmost importance in the process of obtaining many qualities, including color, shape, size, and texture, from photographs of wheat. This process significantly enhances the applicability of these images for classification purposes. An example of the utilization of the Gray Level Co‐Occurrence Matrix (GLCM) technique was observed in the PM, where it was employed for the purpose of feature extraction. Subsequently, a Support Vector Machine (SVM) algorithm was employed for classification. The utilization of this methodology yielded a classification accuracy of 89.23% in the context of wheat stripe rust detection. In (Xie et al. 2016) concentrates on extracting details of wheat stripe rust by applying the use of the GLCM strategy. In the course of experimentation, it was seen that SVM was able to classify the wheat stripe rust illness at 77.78% percent. Relevance Vector Machine on the other hand had a higher classification accuracy of 88.89% of the same task. Successfully utilized machine learning models including SVM, Artificial Neural Networks (ANN), and decision trees in order to precisely identify different wheat infection diseases (Römer et al. 2011). It involves immediate learning of pictures of wheat at a wavelength of 370–800 nm. It is worth mentioning that SVM exhibits superior performance in terms of classification accuracy (93.05%) compared to decision trees and ANN while utilizing different wavelengths. Successfully employed a random forest classifier to accomplish the task of binary classification between wheat stripe rust and healthy wheat plants (Su et al. 2018). The examination of precision, recall, and accuracy metrics is crucial to the evaluation of binary classification. Significantly, within the set of performance metrics, the accuracy parameter (89.4%) exhibits outstanding outcomes in the classification of wheat YR diseases. However, Wang et al. (2015) employed an analysis of canopy layers to categorize YR and BR diseases. The categorization of these rust illnesses is achieved by the utilization of SVM and KNN methods. The experimental results indicate that the KNN algorithm achieves a higher accuracy rate of 92% compared to the SVM algorithm, which achieves an accuracy rate of 68.67%. These findings are based on the utilization of Hyperspectral pictures at the canopy level for the purpose of identifying rust infections.

### 2.2 Deep Learning Methods

**FIGURE 1:**
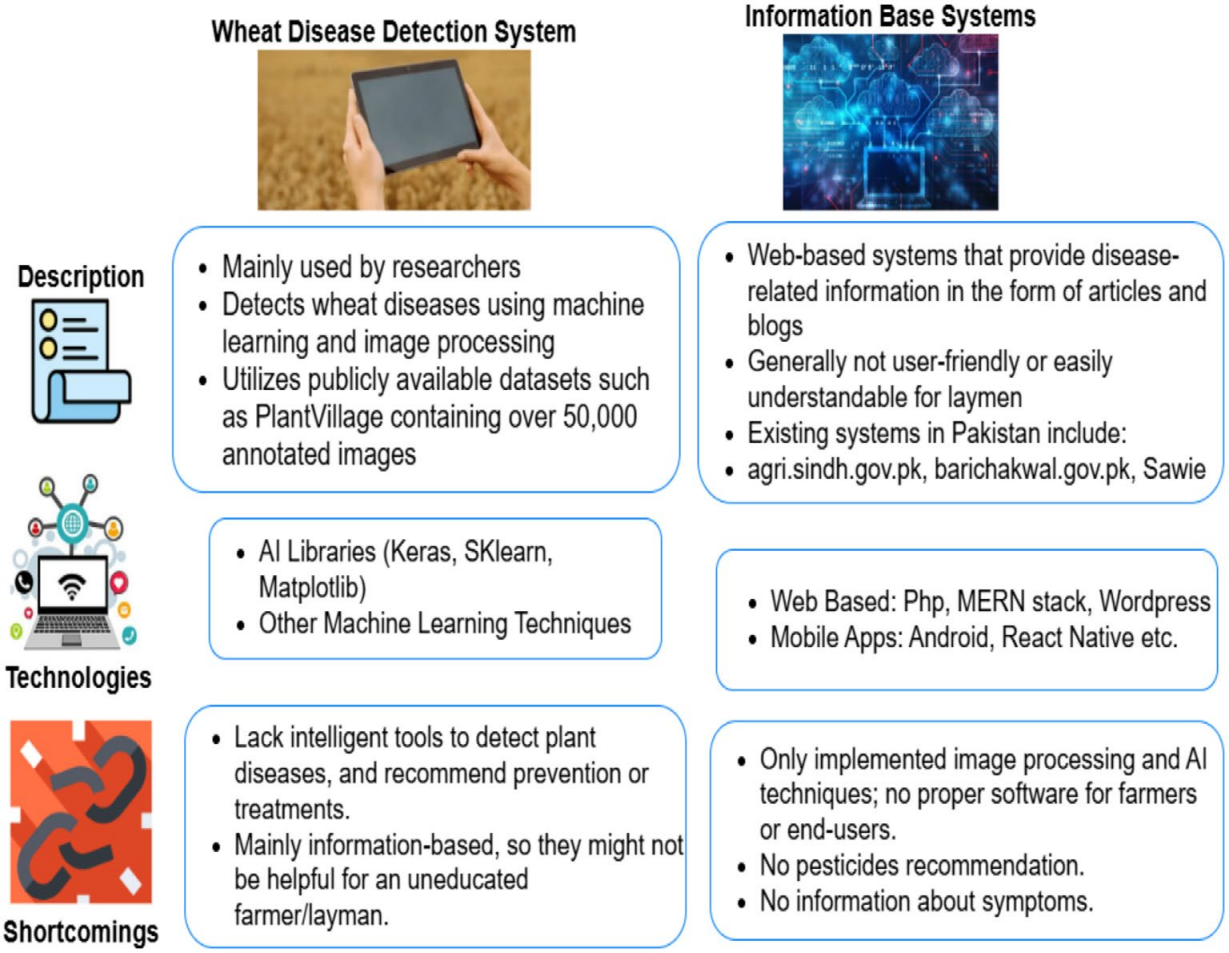
Comparison with existing techniques.

The study conducted by (Kukreja and Kumar 2021) explores the utilization of DLM for automated feature extraction and classification in photos. Numerous researchers have employed diverse DLM to categorize agricultural diseases. Consequently, DPA facilitates automated identification of crop diseases. The study of (Majumdar et al. 2015) proposed a web‐based system for the purpose of crop disease identification. The web‐based solution utilizes an image analyst, feature extractor, and classifier. To extract the key elements of an image, feature extraction methods like c‐means clustering are used. These retrieved features are subsequently utilized as input for the ANN classifier. The ANN achieves a level of accuracy of 84.8%. The ANN attains an accuracy level of 84.8%. Mi et al. (2020) conducted a classification analysis on different types of wheat illnesses. A comparative analysis is performed, wherein pre‐trained models like Alexnet, Resnet‐50, Sequential CNN, and VGG‐16 models are utilized for the purpose of classification. Significantly, by employing these pre‐trained models, the Sequential CNN model attains an exceptionally high level of classification accuracy, reaching 97.20%. In the domain of wheat disease classification, the performance of this model surpasses that of Alexnet (95.10%), Resnet‐50 (97.12%), and VGG‐16 (96.72%) pre‐trained models in terms of accuracy. In addition, the research also investigates the categorization of wheat PM disease by the utilization of a normalized CNN methodology. The efficacy of RNN and Deep Neural Networks (DNN) models in predicting wheat diseases through the analysis of hyperspectral pictures. The findings indicate that the RNN model demonstrates a higher level of accuracy in predicting wheat diseases, surpassing the DNN model by a margin of 0.067 (Pryzant et al. 2017). A Deep CNN (DCNN) was employed in order to detect and classify YR disease. This was achieved by analyzing high‐resolution hyperspectral pictures. The aforementioned photos were acquired utilizing hyperspectral sensors that were installed on a DJI S1000 unmanned aerial vehicle (UAV) platform. Furthermore, researchers conducted a comparative analysis between the DCNN methodology and the inception‐Resnet model in order to classify wheat YR diseases. Therefore, the utilization of the DCNN methodology, which integrates the inception‐Resnet layer, demonstrates proficiency in the identification of wheat diseases within a Three‐Dimensional (3D) setting (Zhang et al. 2019). The breadth of our suggested model is determined by comparing existing methodologies, as discussed in the associated work section depicted in Figure 1.

## 3 Proposed Model (PM)

**FIGURE 2:**
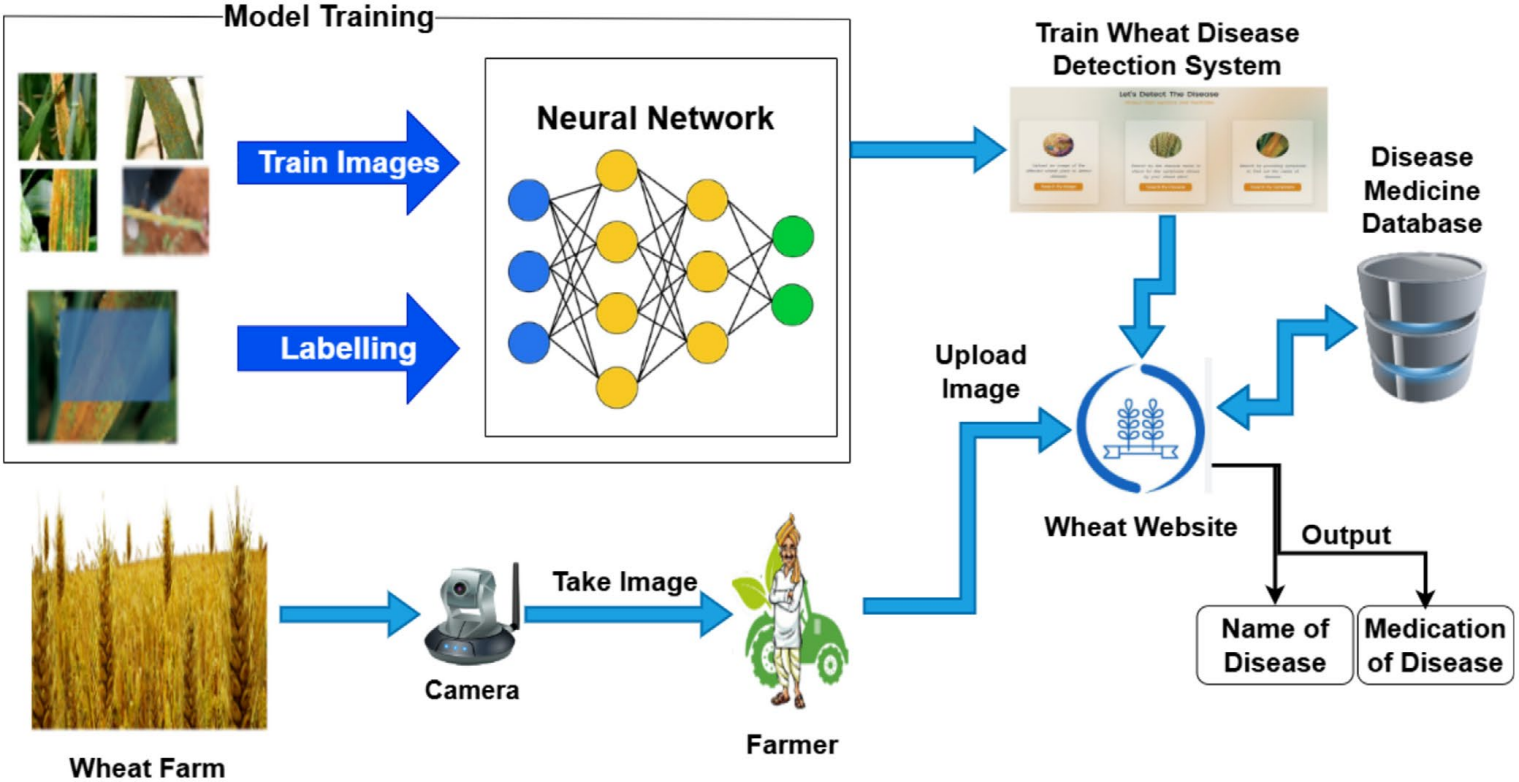
Proposed model.

Figure 2 illustrates PM for wheat diseases identification and recommendation system. The PM divides into different steps such as input data, model training, upload image to detect diseases and treatment recommendation which are explained in detail in subsections of this section.

**FIGURE 3:**
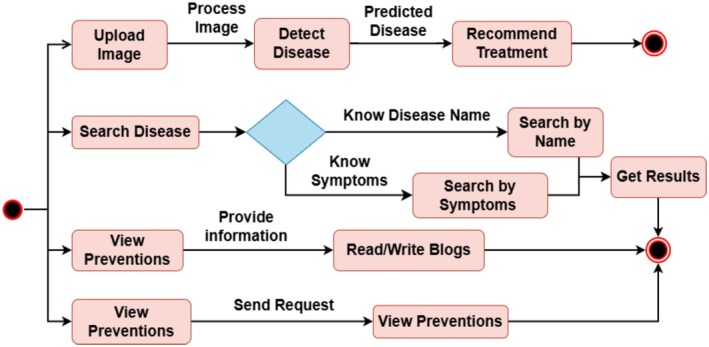
Flow diagram of PM.

The workflow of our application is shown in Figure 3. In general, it is divided into four sections: uploading an image, looking up diseases, reading up on prevention measures, and consultation with a botanist. A trained PM is used to detect wheat diseases from user‐uploaded images. Users of the “search diseases” function can look up diseases by their names or symptoms and receive relevant results. Users can get information to help avoid diseases by posting various vlogs utilizing the application's “view prevention” option. Users can consult a botanist for advice on wheat crop by selecting the “consult botanist” option.

### 3.1 Dataset

**FIGURE 4:**
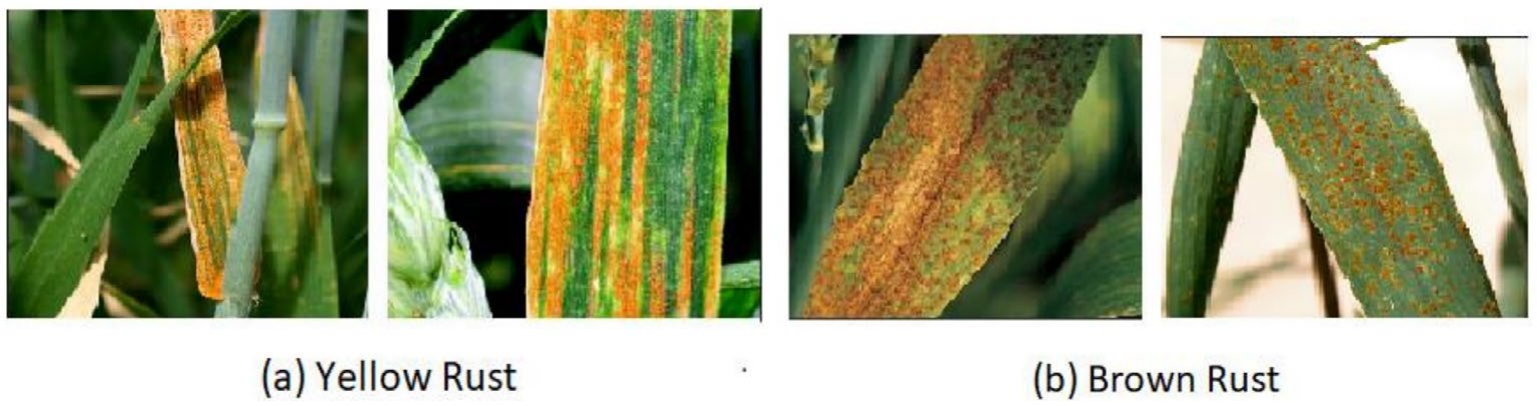
Rust disease samples from dataset.

Two distinct types of wheat rust infections were identified throughout our research: YRand BR. Both healthy plants and plants that had various diseases were photographed (see Table 2 and Figure 4).

**TABLE 2:** A summary of dataset, which encompasses symptoms of diseases along with corresponding number of relevant images.

Class label	Affected region of leaf	Symptoms	Images
YR	Veins of leaf, spikes, and leaf sheaths	Little pustules ranging from yellow to pale orange	1156
BR	Leaf sheath and upper leaf surface	Circular and orange to brown spore pustules	1128
Healthy plant	—	—	1395
Total Images	—	—	3679

The images of YR and BR were taken from a Kaggle dataset, which is available at https://www.kaggle.com/datasets/sinadunk23/behzad‐safari‐jalal. The dataset includes 3679 images divided into three different categories, as shown in Table 2. Three subsets of the dataset were created, with a training and validation split 70:30. When creating machine learning models, previous studies showed that a 70:30 split ratio produced best outcomes (Nguyen et al. 2021; Seidu et al. 2022). The publicly accessible Kaggle dataset used in this research is inherently limited due to the lack of detailed metadata on acquisition. The data on the exact kind of wheat types or the exact field conditions (i.e., date, location, light) that the photos were taken in was just not available in the source repository. This absence of upstream information directly limits our capacity to provide finer labeling on these parameters in Figure 4 and other visualizations of the raw data. We undertake to make sure that all internally produced charts and figures, e.g., model performance (e.g., training history, confusion matrix), are as clear as possible, labeled accurately and with detailed captions.

In this research paper, two forms of wheat rust attacks, YR and BR, were taken into consideration alongside healthy plant samples (see Table 2 and Figure 4). The images were collected as a publicly accessible Kaggle dataset with 3679 images placed in three categories including YR, BR, and healthy. Because the data is community sourced, there is no acquisition metadata (such as variety of wheat and field conditions), which is a weakness of this research and why Figure 4 lacks such labelling. To come up with the model, the dataset was divided into the training and validation subsets in the ratio of 70:30 following the earlier studies that had provided positive outcomes using the same ratio. However, this was not accompanied by k‐fold cross validation, which can be viewed as restricting the model to predicting the unseen dataset. Boosting the model's robustness and reliability in future work will be achieved through resampling with the incorporation of cross validation and other resampling methods.

This study considered two types of wheat rust infections, YR and BR, in addition to healthy plant samples (see Table 2 and Figure 4). The pictures were gathered in the form of a publicly available Kaggle dataset, which has 3679 pictures divided into three categories, including YR, BR, and healthy. Since the dataset is community sourced, there is no detailed acquisition metadata (including wheat variety information and field conditions), which is a limitation of this study and the reason why Figure 4 does not have such labeling. To develop the model, the dataset was separated into the training and validation parts at the percentage of 70:30 in line with the previous research that demonstrated good results with such a partitioning method. Nevertheless, there was no k‐fold cross validation, which can be considered limiting the model to generalize to unseen datasets. Resampling will be used to boost model robustness and reliability in future work by including cross validation and other methods to resample.

Our proposed methodology consists of two main steps. In the first step, images are classified to determine the class of diseases they belong to, such as BR/YR, or a healthy plant. In the second step, diseases are identified based on their symptoms, and appropriate treatments are suggested accordingly. Moreover, if the user enters the name of a disease, symptoms are provided, and treatment is also suggested. The web‐based application is integrated with a trained model capable of detecting wheat diseases using both images and symptoms. The application offers treatment recommendations for identified diseases based on the provided input.

### 3.2 Wheat Diseases Detection

**FIGURE 5:**
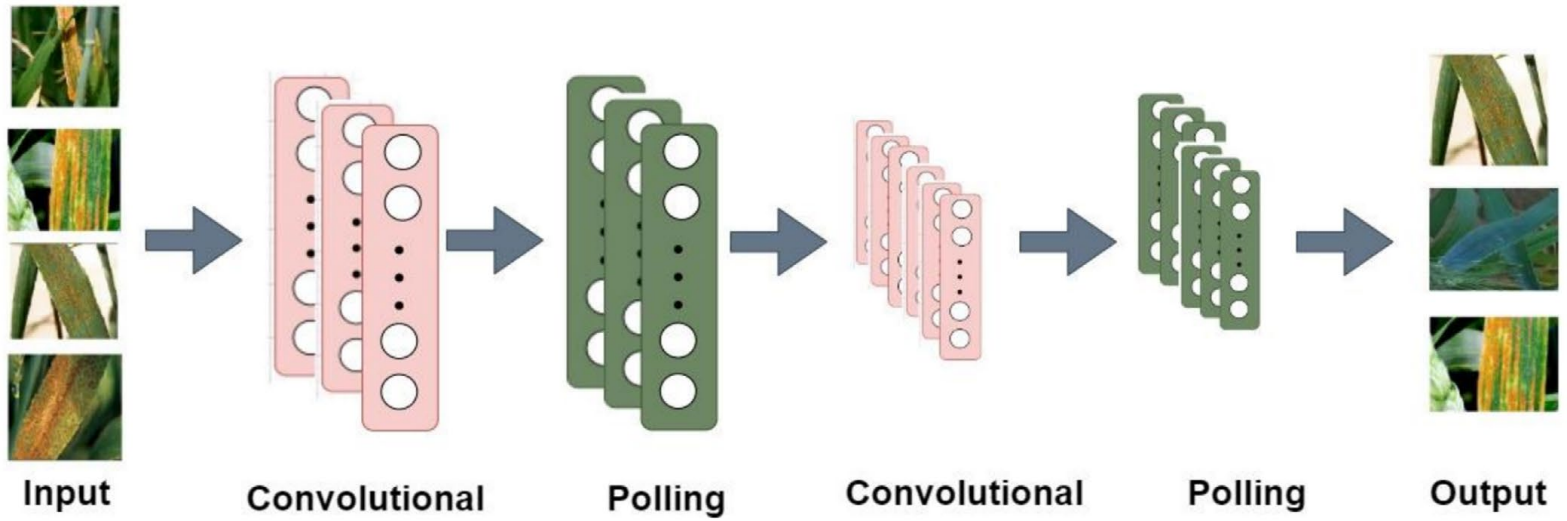
CNN.

Figure 4 depicts the proposed paradigm for wheat DD. The training dataset is sent to DLM, which learns distinguishing features of three distinct data classes (see Table 1). The weights (parameters) of the model are changed in the backward run to back‐propagate the error determined using the anticipated and expected values. The training procedure is repeated a predetermined number of times. After training is complete, the model's accuracy is evaluated. The PM for wheat DD is depicted in Figure 5. When convolution, pooling, and regularization are incorporated into the signal processing pipeline, CNN can effectively address the problems of image classification. The proposed model was trained using an extensive wheat disease image database. Once trained, the model is able to take an input image and make precise classifications, labeling the image as belonging to a YR, BR, or healthy wheat.

Training requires a 256‐by‐256‐pixel RGB image. The model uses convolutional layers with a 3 × 3 kernel size. Maximal pooling is employed to reduce the training vector space. The CNN which consists of 6 convolution layers, 6 max‐pooling layers, and one fully connected layer as shown in Figure 5, is proposed for wheat DD. Rectified Linear Unit (ReLU) is employed in the convolutional layer. The ReLU serves as an activation function in numerous layers. Following the single connected layer is a SoftMax classifier. Utilizes Adam optimization. The utilization of one‐pixel Convolution cadence. The weights of only convolution and fully connected layers can be trained. The input image's size is reduced by the max pooling layer, and softmax makes the final determination. Dense layers for learning and prediction are placed after the convolution and pooling layers. The activation function that normalizes the distribution of K composite functional concatenations is utilized at the conclusion of the Dense layer SoftMax. Each layer of nodes is trained using the output of the previous layer. Consequently, nodes in each succeeding layer are able to distinguish increasingly complex and specific characteristics.

### 3.3 Wheat Diseases Treatment Recommendation

**FIGURE 6:**
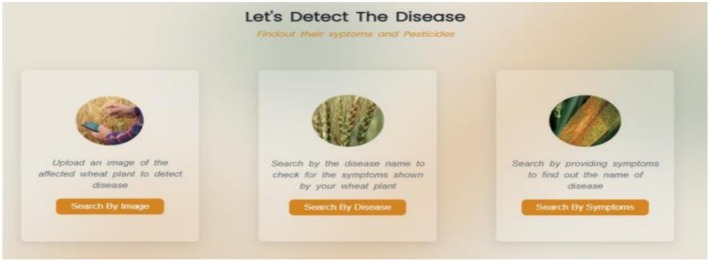
Screenshots of three options to detect disease.

**FIGURE 7:**
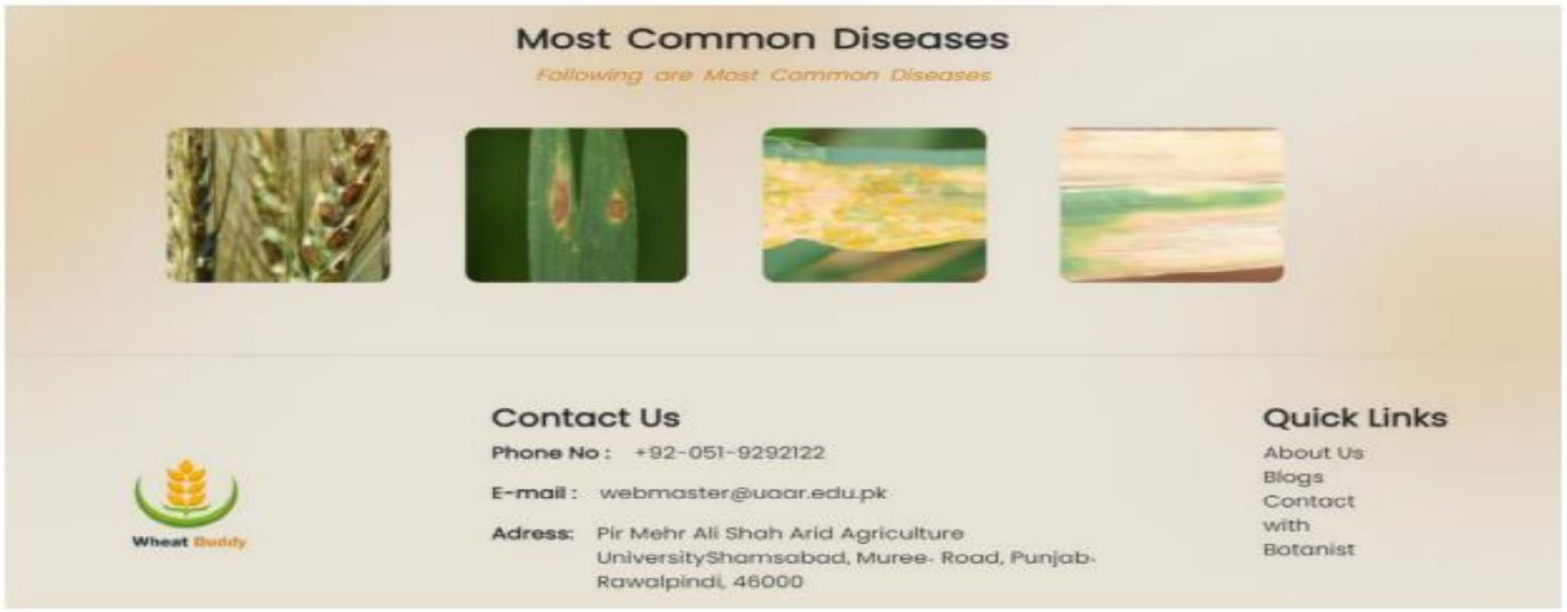
Screenshots of most common diseases.

**FIGURE 8:**
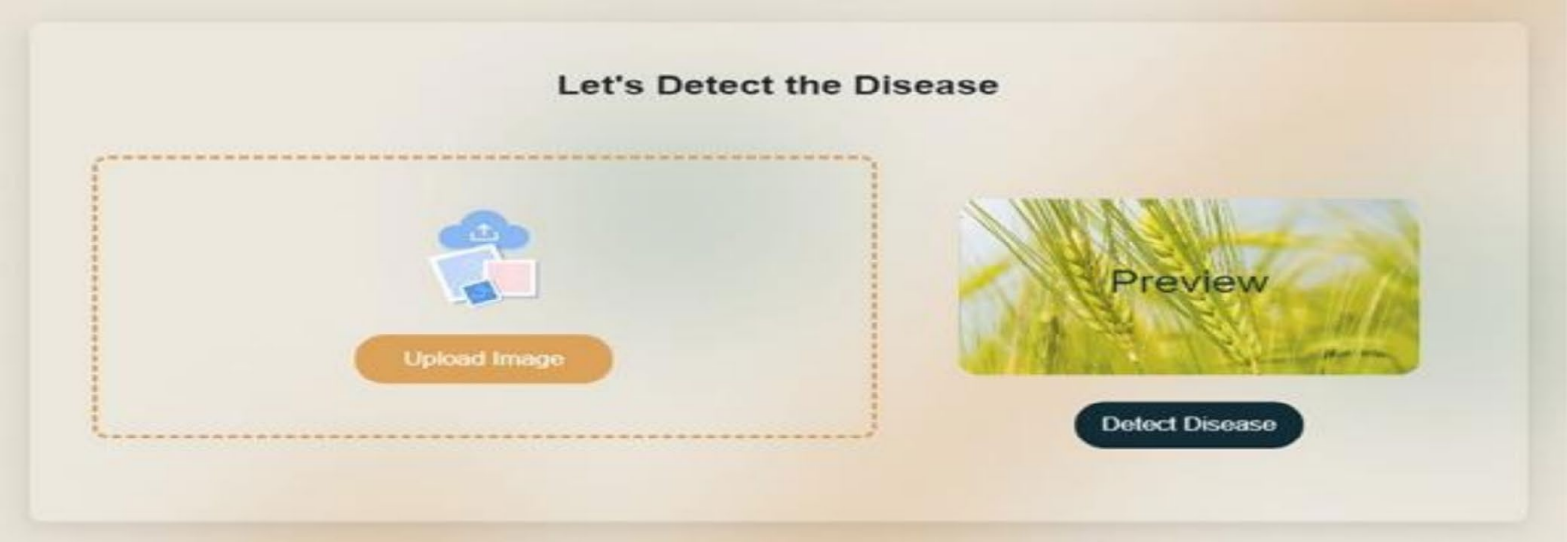
Screenshots of wheat DD and treatment recommendation: Detect diseases by Image.

**FIGURE 9:**
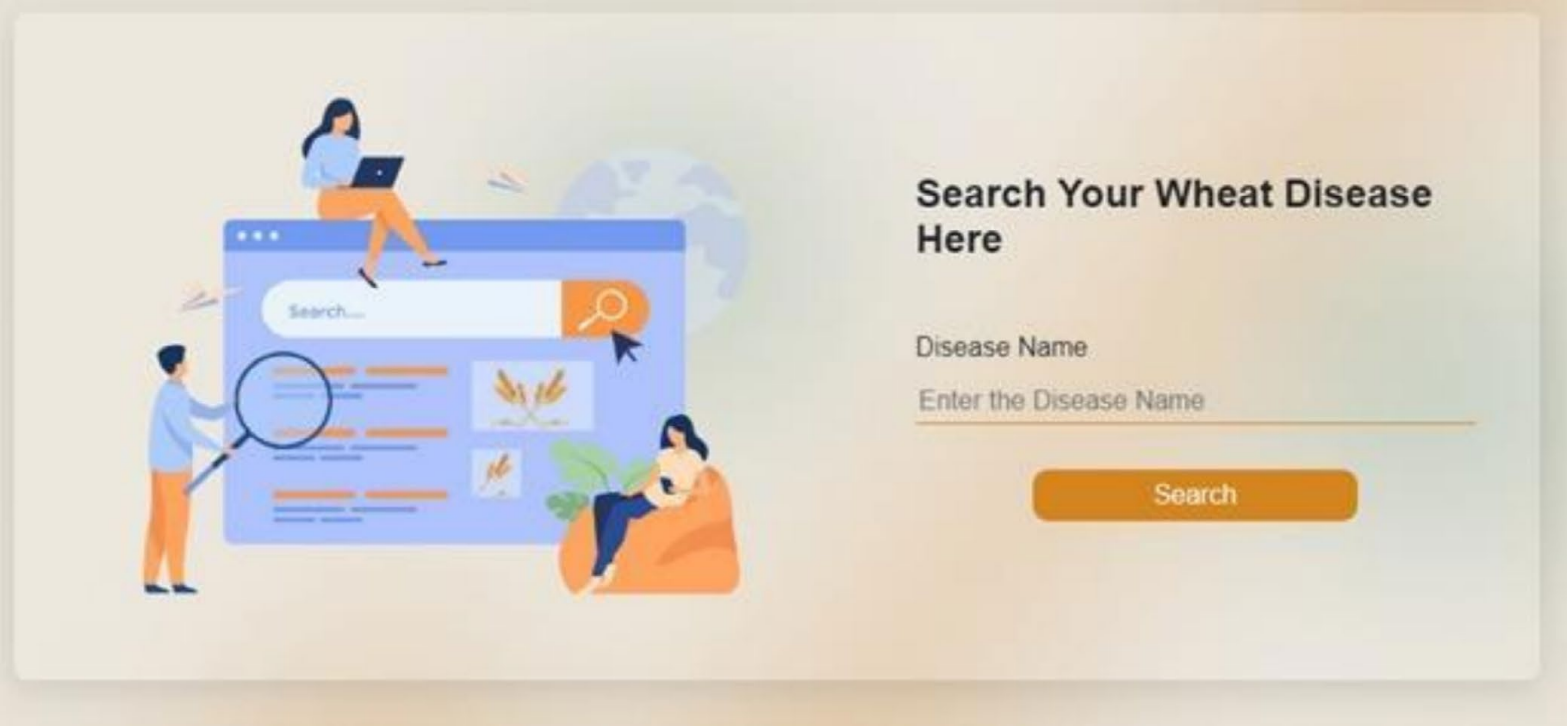
Screenshots of wheat DD and treatment recommendation: Recommend treatment of diseases.

**FIGURE 10:**
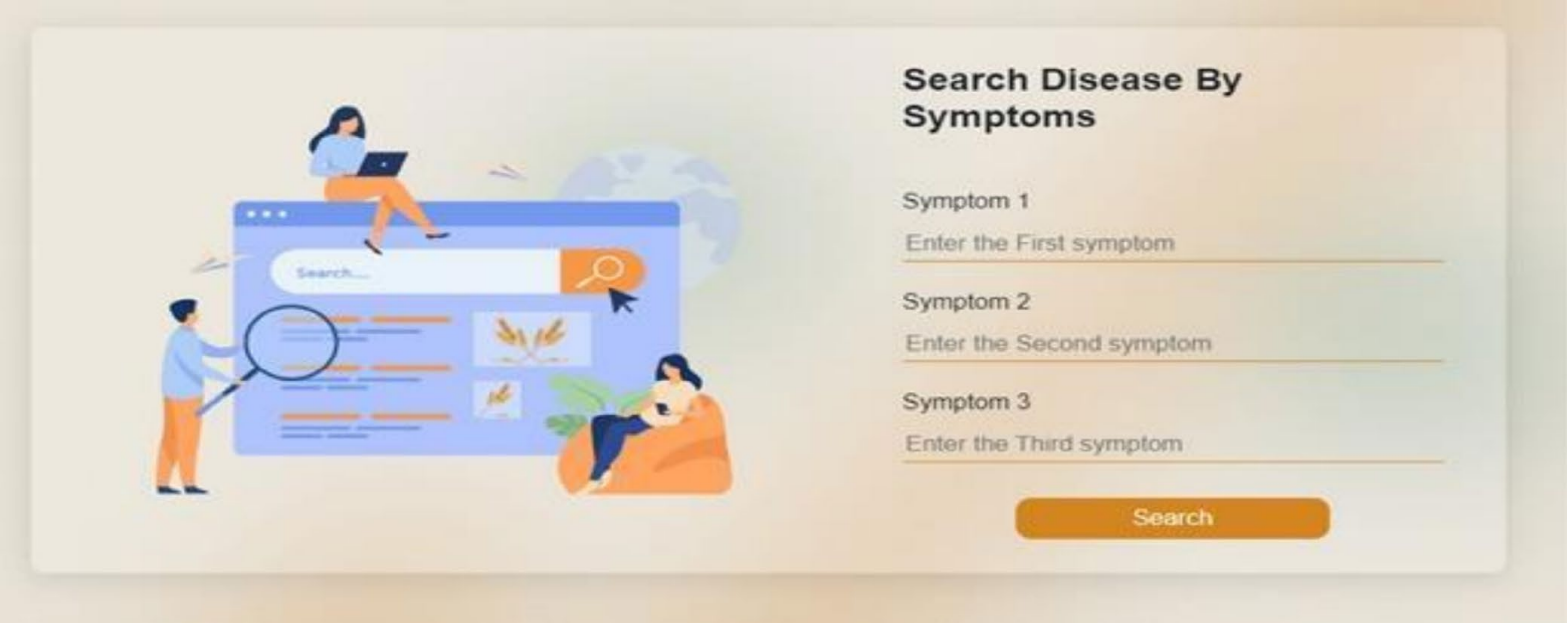
Screenshots of wheat DD and treatment recommendation application: Detect diseases by symptom.

The wheat diseases treatment recommendation system operates by receiving the name of a wheat disease and subsequently providing suggestions for appropriate medications. The medication or treatment of different wheat diseases such as PM, SLB, TS, HB (Scab), LS, KB, RR, LF, BYDV, WSBMV, and Wheat WSMV are suggested by the system. We implemented regular expressions to extract names of wheat diseases from user inputs provided via the front‐end interface. These terms were carefully crafted to reflect differences in disease names and make proper identification. The recommendation system was created on the basis of REST API architecture. Based on this structure, the system takes disease names as input queries and uses an algorithm to align each disease to the appropriate medicinal recommendations. The system of recommendations was perfectly incorporated into the front‐end interface and provided a user‐friendly and intuitive experience. The user can also enter the name of a wheat disease and the system, based on that, will provide the available treatment options. The process of integration makes sure that there is real‐time interaction between the recommendation system and users. For user data protection, the system is designed to process uploaded images and input queries in a non‐persistent manner; all images are discarded immediately after classification, and user queries are anonymized for system logging, ensuring the security and privacy of sensitive agricultural data. Figures 6, 7, 8, 9, 10 depict the user interface of the web‐based application.

## 4 Results and Discussion

**FIGURE 11:**
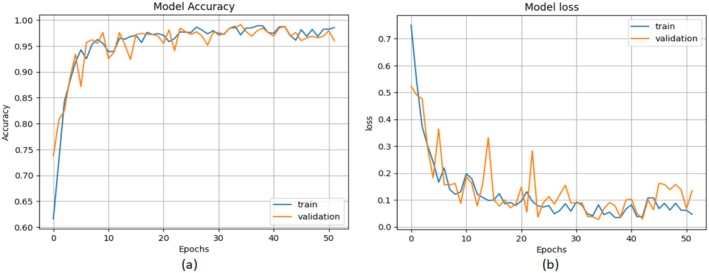
Model performance (a) accuracy graph (b) loss graph.

To assess effectiveness of suggested model, dataset was categorized as; train, validate and test. 30% of the data was used for testing, while the remaining 70% was used for training and validation. Figure 11 depicts model performance in terms of accuracy and loss curves for classifying photos into accurate classes. If training and validation accuracy curves increase gradually with no discernible variations, a model is deemed to be good and useful (Hossin and Sulaiman 2015).

**FIGURE 12:**
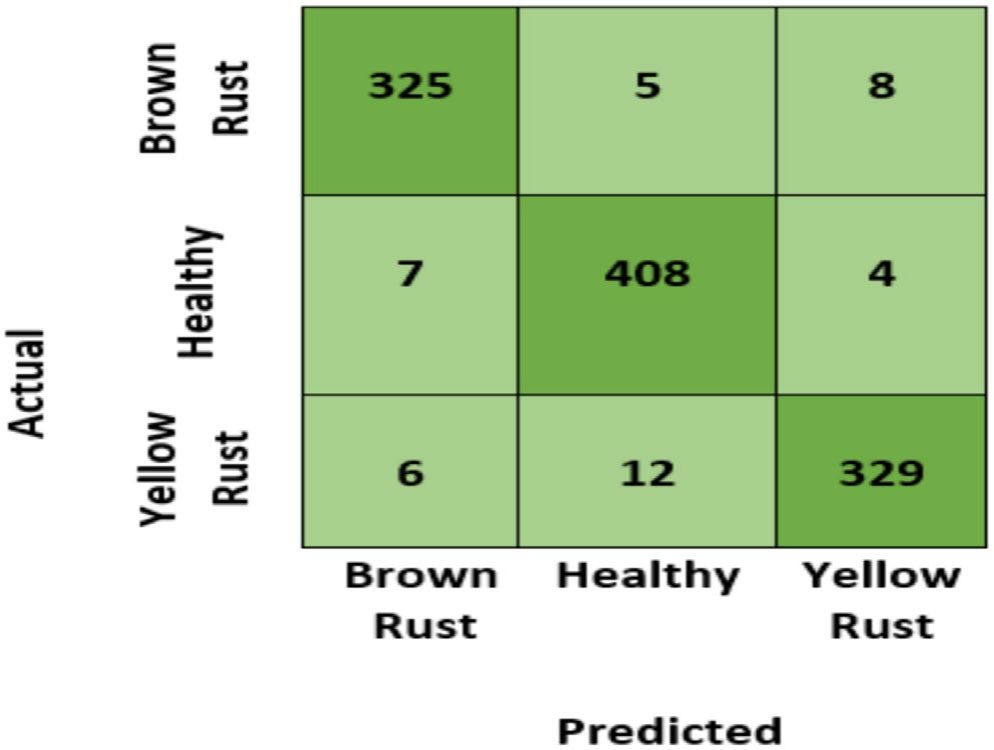
Confusion matrix.

**FIGURE 13:**
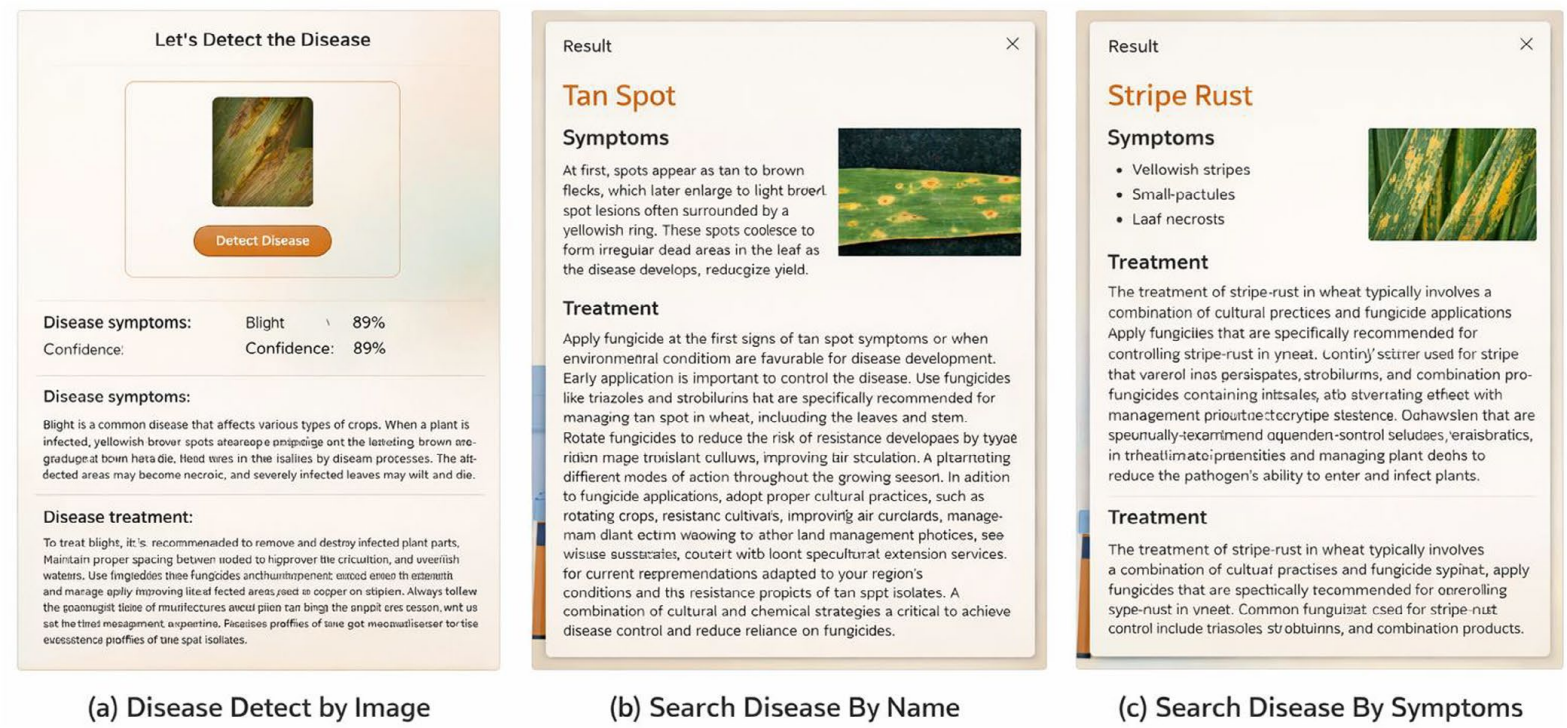
Screenshots of wheat DD and treatment recommendation output.

In order to have a thorough evaluation of the model performance other than the recorded overall accuracy of 96%, it is important to delve deeper with the help of the confusion matrix (Figure 12). The diagonal lines in the matrix validate a high success rate in classifying all the three types of YR, BR, and Healthy plants. But a more precise misclassification analysis is given by looking at the elements of the off‐diagonal. We have found that most misclassification appears between the two disease classes (YR and BR) where the model is at times confusing one type of rust to another. This implies that the fine visual characteristics that differentiate YR pustules and BR pustules pose the most challenge to the classifier, which is a typical problem in the diagnosis of plant pathology. Incorrect classification of a diseased and healthy plant happens less often. This step‐by‐step breakdown makes certain that the strengths of the system are reported correctly and a clear discussion of the current diagnostic limitations of the system is also discussed. In addition, we utilized confusion matrix, precision (P), recall (R), and F1‐score using Equations ((1), (2), (3)) to evaluate diagnostic accuracy of PM for WPD. Figure 12 illustrates confusion matrix. The diagonal values of perplexity matrix represent number of correct answers that match validation data. The off‐diagonal numbers depict number of incorrect predictions made using validation data. The confusion matrix demonstrates that PM can reliably predict both healthy and damaged images of wheat leaf. When suggested model is utilized, Table 3 displays P, R and F1‐score for each class.

**TABLE 3:** Accuracy measures for proposed model on wheat diseases detection dataset.

Classes	P	R	F1 score
YR	0.965	0.948	0.956
BR	0.961	0.961	0.961
Health plant	0.96	0.974	0.966

P is evaluated relative to the model's predicted outcomes. The model predicts a number of genuine and accurate samples. P is the proportion of correct and actual samples among all identified correct samples. R is based on samples that are fairly accurate. R is defined as the proportion of correct and actual samples among all correct samples that are identified. The score F1 is calculated as the weighted harmonic mean of P and R. This score takes False Positives (FP) and False Negatives (FN) into account. Where TP and TN are true positives and negatives, respectively. It conveys the equilibrium between P and R. Figure 13 depicts the output of the application.

(1) $$P=\frac{\mathrm{TP}}{\mathrm{TP}+\mathrm{FP}}$$

(2) $$R=\frac{\mathrm{TP}}{\mathrm{TP}+\mathrm{FN}}$$

(3) $$F1\;\text{Score}=2\times \frac{R\times P}{R+P}$$

The confusion matrix of the proposed model can be seen in Figure 12. The number of correctly classified samples is shown by the diagonal elements, and the misclassifications are shown by the off‐diagonal elements. The study of these misclassifications indicates that the majority of errors were made between these two disease groups (YR and BR), and less between diseased and healthy samples. This misclassification behavior is anticipated in deep learning models as visual features of a disease look similar, particularly at the initial stages of the disease.

In general, the confusion matrix shows that the proposed model is effective in classifying healthy and diseased wheat leaves. Table 3 gives *P*, *R*, and F1‐score of models by class, giving a more detailed assessment of model performance than accuracy. Precision is the fraction of the correctly predicted positive samples of all predicted positives and recall is the fraction of the correctly identified positive samples of all the actual positives. F1‐score is the harmonic mean of both precision and recall, which balances the metrics, with the consideration of FP and FN. This thorough research supports the conclusion that the model is able to give efficient classification across classes.

Figure 13 also shows the output produced by the application and it indicates that it is able to produce correct disease detection results in practice.

### 4.1 Comparative Analysis Based on Existing Studies

The research uses a CNN for the detection of wheat diseases and embeds it in a web application. Unlike previous works, this research values simplicity and computational efficiency over using much deeper models like ResNet or Inception. Deeper models, while having higher accuracy owing to their ability to capture more sophisticated patterns, come at a much higher computational cost and are often avoided in resource‐constrained environments. On the other hand, the simpler CNN model used in this research still has a respectable 96% accuracy (See Table 4), indicating its effectiveness for this particular task.

**TABLE 4:** Comparative analysis table.

Study	Model used	Accuracy (%)	Platform	Scope	Treatment recommendation
PM	CNN (custom 6‐layer)	96.0	Web application	Yellow rust (YR), brown rust (BR), healthy plants	Yes
Mi et al. (2020)	ResNet‐50, AlexNet	97.2 (ResNet‐50)	Mobile app	Spot blotch (SB), YR, black rust, leaf spot	No
Römer et al. (2011)	SVM, ANN, Decision Trees	93.05 (SVM)	N/A	Brown rust, healthy plants	No
Zhang et al. (2019)	DCNN, Inception‐ResNet	85.0 (DCNN)	UAV images	Yellow rust	No
Xie et al. (2016)	GLCM + SVM, RVM	88.89 (RVM)	Mobile app	Yellow rust, powdery mildew	No
Majumdar et al. (2015)	ANN (C‐means clustering)	84.8	Web application	Snow molds, yellow rust, septoria leaf blotch, powdery mildew	No

The PM integrates its trained model into a web‐based interface for disease detection and treatment recommendations. While this is user‐friendly, there might be some problems with accessibility in areas with poor internet connectivity. There are some mobile‐based implementations that provide offline functionality, representing a trade‐off between accessibility and computational capability.

The design decision of a simple Convolutional Neural Network (CNN) architecture was based on the main goal that the developed system should be sustainable and should operate within the constraints of the resources available on a web‐based system to be deployed in a resource‐limited setting. Whereas the authors recognize that more current models like ResNet, Inception, or VGG may be slightly more accurate in classification, initial comparative studies showed a dire trade‐off: these more complex models are usually larger by file size and/or by a large degree of inference latency. In a real‐time application, users might be limited by bandwidth or low power; hence, the overhead of a massive model to deployment jeopardizes the sustainability of the system and user experiences. The model that has been chosen is a good tradeoff, with a 96% accuracy on the test set, but with a small computational footprint that would scale well to being deployed effectively to the Web. However, the authors also concede that the future work ought to focus on the relocation of optimized and complex architectures (e.g., lightweight versions such as MobileNet or fine‐tuned counterparts of ResNet) as long as the benefits in accuracy can be achieved without compromising the existing real‐time performance properties of the system.

- Model Performance: This work demonstrates competition accuracy with the more advanced models such as ResNet‐50 (97.2%). However, a less complex CNN is employed to lower the cost of computation, which can be deployed in real‐time over the web.
- Platform diversity: Unlike most of the studies where only a single approach is studied, such as mobile application stand‐alone or UAV‐platform‐centric, the study at hand incorporates its model into a web‐based system where the aspect of accessibility is focused on by using online services.
- Comprehensiveness: The study adds to its functionality by incorporating treatment recommendations, which many comparative studies lack.
- Application Context: Mobile and UAV platforms from other studies offer offline and high‐resolution capabilities but may require more resources to run; therefore, constraining their scalability.

### 4.2 Threats to Validity

#### 4.2.1 Internal Validity

- Model Generalization: The study is using a CNN model trained on a dataset taken mainly from Kaggle. If such a dataset does not completely represent the variety of wheat rust infections—say, variations in lighting, leaf age, and disease stages—the model might overfit to the dataset and poorly generalize to real‐world data.
- Data Partitioning: The training‐validation splitting is 70:30, which means that the dataset of all the disease classes is well‐balanced. Nonetheless, the presence of any imbalance (e.g., more images of healthy plants) would distort the results. Additionally, the model is not cross‐validated and it is simply trained using one 70:30 split only. This is a drawback in that it gives the model a higher chance of overfitting to the particular training data and performing poorly on completely unknown, out‐of‐sample data, hence limiting its ability to generalize.
- Confounding Factors: Environment or image‐capture conditions (e.g., shadowing or overlapping leaves) can be a source of noise, which inaccurately influences the model. This is further complicated when the model is taught on clean and curated samples and increases the chances of poor‐quality or noisy field images with different angles and resolutions being misclassified.

#### 4.2.2 External Validity

- Dataset Limitations: The dataset used appears to be comprehensive, but the real‐world applicability depends on whether images from different geographical regions, wheat varieties, and disease manifestations were included.
- Scalability of the System: Web application deployment assumes stable internet connectivity for the end users; that is, the farmers. This may limit its efficacy in rural areas where connectivity is sparse or unreliable.
- Disease Expansion: The system is tailored for YR and BR, with only minimal inclusion of other diseases such as Wheat Blast and Spot Blotch. The results may not generalize to these diseases without additional training data.

#### 4.2.3 Construct Validity

- Evaluation Metrics: Metrics such as precision, recall, and F1‐score are suitable for this system. However, the absence of field trials with real end‐users on actual farms impels that effectiveness is evaluated only in controlled environments.
- Manual Labeling: The mislabeling of images in the dataset can introduce noise, reducing the reliability of the trained model.
- The existing performance indicators (e.g., 96% accuracy) of this model are obtained on the basis of in silico testing on a publicly obtained, controlled dataset only. The authors clearly admit that there has been no validation of the same in a real farm setting so far. This is a major drawback, since the controlled laboratory setting cannot completely replicate practical constraints of the agricultural environment, including variable natural illumination, camera shake, overlapping leaves, or the high noise levels needed by the end‐user. Therefore, the stated accuracy is the level of the model performance in the ideal conditions and not in the real efficacy of the model as a diagnostic instrument. The future work is urgently required to implement the system in field tests and define its external validity and real applicability by farmers.

#### 4.2.4 Conclusion Validity

- Bias in Results Interpretation: Reporting an impressive accuracy (96%) might not fully capture model limitations or challenges faced during testing. Overemphasis on quantitative results could lead to overlooking qualitative insights, such as farmers' ease of use or model explainability.
- Treatment Recommendations: The recommendations provided by the system are based on fixed rules and might not be flexible enough to apply in unforeseen situations, including combined diseases or diseases under certain environmental conditions, which restricts its applicability in most complicated real world situations.
  The existing treatment recommendation module is developed as a rule‐based system that gives instant and interpretable guidance, depending only on the type of rust that is identified (YR or BR). Although such a method makes sure that the recommendations on individual illness cases are fast, actionable, and consistent, the authors admit that such a method has a fundamental limitation of the lack of flexibility needed to apply such recommendations to real‐life agricultural conditions. More precisely, the module fails to incorporate the factors including local environmental conditions (e.g., humidity, temperature) or the occurrence of pathogen diseases (co‐infection with other pathogens) currently. The advice then offered might not work particularly well in highly nuanced scenarios, which is an obvious drawback on the overall practical usefulness of the system and needs further refinement before it can really be used in precision farming.

## 5 Conclusion and Future Work

A web‐based application for the categorization of wheat rust infections and treatment advice of wheat diseases is proposed in PM work. A DLM, such as CNN, was utilized to classify wheat rust infections. Nonetheless, rust, a common disease that affects wheat crops, can wipe out entire harvests. Thus, in order to heal plants and ensure optimal yields, it is critical to diagnose and address these diseases as soon as possible. The experiment was done on Google Colab using the Python programming language. For the training and testing model, 3679 images of healthy plants, BR, and YR were employed. With optimizer = “Adam”, batch size = 32, and epochs = 50, the accuracy rate was 96.01%. Furthermore, this application will aid farmers in disease detection, crop protection, and disease treatment recommendations. Our next effort will entail broadening classification to cover more wheat diseases in order to improve the usefulness of our web‐based application. Furthermore, this work might be improved by applying a variety of feature detection approaches to reduce data noise. Furthermore, a wide range of DLM, including but not limited to DCNN, VGG16, Residual Neural Network, YOLO, and others, can help refine this work. The modular CNN architecture is flexible by nature with regard to scaling to more complex architectures. There will be three main directions to work at in the future. Initially, the detection capabilities of the system will be extended to the detection of other wheat diseases including Wheat Blast, Spot Blotch, etc. which will necessitate the gathering and curation of larger, labeled datasets. Second, the model will be introduced and tested in real‐life farm settings to confirm its practicability and to optimize the logic of treatment recommendation according to the circumstances in the field. Lastly, more sophisticated deep learning models, including ResNet and Inception, will be investigated to increase the level of accuracy and make sure that the computational costs are still affordable to implement in practice. Further growth is obviously geared towards the development of its diagnostic spectrum to cover other disorders, such as Wheat Blast or Spot Blotch. The main needs associated with this expansion are to gather new, annotated image information to retrain the current network and therefore improve the usefulness of the system.

## 6 Developments in Generative AI and AI‐Assisted Technologies in Manuscript Preparation

When writing this manuscript, the authors assure that they did not engage in writing, content generation, image creation, and data collection and analysis with the help of a generative AI or AI‐assisted technology. The human authors did all the research, writing, and analysis. Had they applied any AI tools, they would have outlined their application very clearly in the Materials and Methods section as per editorial standards.

## Author Contributions

**Nergis Gulzar Abbasi:** conceptualization (equal), formal analysis (equal), methodology (equal), writing – original draft (equal). **Aiman Khan Nazir:** formal analysis (equal), methodology (equal), supervision (equal), writing – original draft (equal). **Hamza Iqbal:** data curation (equal), formal analysis (equal), methodology (equal), software (equal), validation (equal), writing – original draft (equal). **Atif Ali:** software (equal), writing – review and editing (equal). **Sadia Ali:** data curation (equal), investigation (equal), software (equal), writing – original draft (equal). **Sagheer Abbas:** formal analysis (equal), methodology (equal), validation (equal), visualization (equal), writing – review and editing (equal). **Muhammad Adnan Khan:** formal analysis (equal), software (equal), supervision (equal), validation (equal), writing – review and editing (equal).

## Funding

The authors did receive any specific funding for this study.

## Ethics Statement

The authors have nothing to report.

## Consent

The authors have nothing to report.

## Conflicts of Interest

The authors declare no conflicts of interest.

## Data Availability

The data used to support the findings of this study are available from the corresponding authors upon request.
